# Cochlear implant therapy improves the quality of life and social participation in the elderly: a prospective long-term evaluation

**DOI:** 10.1007/s00405-023-08443-6

**Published:** 2024-02-14

**Authors:** Christian Issing, Andreas G. Loth, Kenan D. Sakmen, Leon Guchlerner, Silke Helbig, Uwe Baumann, Johannes Pantel, Timo Stöver

**Affiliations:** 1 Department of Oto-Rhino-Laryngology, Goethe University Frankfurt, University Hospital, Frankfurt/Main, Germany; 2 Working Group On Geriatric Medicine Specializing in Psychogeriatric Medicine and Clinical Gerontology, Institute of General Practice, Goethe University Frankfurt, Frankfurt/Main, Germany

**Keywords:** Cochlear implant, Quality of life, Elderly, Older patients, Long-term results

## Abstract

**Purpose:**

In recent years, the number of elderly cochlear implant (CI) candidates is continuously rising. In addition to the audiological improvement, other positive effects of CI treatment can also be observed in clinical routine. The “quality of life” as a parameter of success directly experienced by the patient is increasingly becoming the focus of clinical research. Although there are already clear indications of a rapid and significant improvement in quality of life, there is still a lack of systematic, prospectively collected longitudinal long-term data in patients over the age of 65.

**Methods:**

This prospective longitudinal observational study included 31 patients between the age of 71 and 92 years who had first been treated unilaterally with a CI 6 years ago. In addition to free-field monosyllable recognition, quality of life was assessed using the World Health Organization Quality-of-Life Scale-old (WHOQL-OLD). The results were compared with the data from our previous study, in which we focused on the short- and medium-term effects on quality of life. In both studies, the same patient population was examined. In addition, these study data were compared with an age-matched average population.

**Results:**

In speech recognition, there was no significant change from the control 6 months postoperatively compared with the results 6 years postoperatively. No significant changes occurred in the total quality of life score or any of the other six facets of quality of life when comparing the results 6 months postoperatively with the results 6 years postoperatively. In “Social participation”, the CI patients even exceed the values of the age-matched average population 6 years after treatment.

**Conclusion:**

Improvement in the quality of life and especially in social participation appears stable over many years in elderly patients after hearing rehabilitation with a CI.

## Introduction

The cochlear implant (CI) has been successfully used for hearing rehabilitation of deaf and severely hearing-impaired patients for over three decades. While initially, only completely deaf patients were treated with a CI, the indication for a CI has been successfully extended [1–3]. Severe hearing loss is one of the most common chronic conditions in the elderly population [4–6]. As a result of demographic change in Europe, the proportion of elderly and very aged CI candidates has been steadily increasing in recent years. With a comparable—but more variable—improvement in speech comprehension compared to younger patients, CI treatment in Germany is offered without an age limit for suitable CI candidates [7–10]. Moreover, an increasing number of studies demonstrate not only the stability of monosyllable recognition but even in many cases, an improvement after more than 1 year postoperatively [11–13]. Especially in elderly patients, further effects beyond the audiological measurable improvements can usually be observed in clinical routine. Due to the specific psychosocial situation of higher age with common loss of social interaction and an increasing physical impairment, this age group benefits from hearing rehabilitation in particular. The influence of hearing rehabilitation on social participation and, therefore, on the quality of life, previously significantly limited by the hearing disorder, is ultimately the CI-treatment success directly experienced by the patient. Consequently, the assessment of the quality of life has developed into an important instrument for evaluating the success of treatment and has now also been implemented in the German Whitebook on Cochlear Implant Care, the German Cochlear Implant Guideline and in the German Cochlear Implant Registry [15–18].

In the literature, there are numerous indications for a rapid and significant improvement in the quality of life in the elderly related to CI treatment. Innumerable studies use questionnaires to assess health-related quality of life, such as the widely used Nijmegen Cochlear Implant Questionnaire. Apart from a lack of validation for elderly patients, a valid comparison with an age-matched healthy general population is impossible. Most studies focus on the short- and medium-term effects of hearing rehabilitation on the quality of life [19–25]. Consequently, the aim of this prospective longitudinal study was to observe the same patient population several years after CI implantation and to compare the study cohort with an age-matched healthy general population.

## Patients and methods

### Study design

This prospective long-term study is a follow-up to our previous study (Issing et al. [19]). For the initial study, candidates were recruited at our institution from the third quarter of 2015 until the third quarter of 2017. For the presented study, patients already recruited into the initial study of 2020 were followed up at annual checkups about 6 years postoperatively after initial unilateral CI treatment. Data for this study were collected from the second quarter of 2022 through the first quarter of 2023. The data from this study were compared with the results of our initial study (Issing et al. [19]). The study was conducted in accordance with the Declaration of Helsinki 1964, and with approval of the local ethical review committee (105/15). All patients gave their written informed consent for inclusion before they participated in this study.

### Patients

Thirty-one patients (12 men and 19 women) of the 34 patients (13 men and 21 women) initially 2020 recruited could be included in this study. One patient was excluded as the CI had to be explanted due to implant infection; two patients could not participate in the survey due to health problems not related to the CI.

### Freiburg monosyllabic speech test (FMS)

Audiological data collected during the 6 year CI aftercare as part of routine clinical practice were analyzed. The Freiburg monosyllabic speech test (FMS) at 65 dB SPL was used to evaluate the monosyllable recognition in the free field. For the continuous monitoring of monosyllable recognition over the years, the ear (first) treated with a CI was continuously evaluated with the FMS, and the other ear was masked by broadband noise or mechanical blocking.

### Quality of life assessment

Our study aimed to assess the quality of life over the years in patients who had been treated with a CI for the first time. To be able to compare the results with our previous study, we used the German version of the World Health Organization Quality-of-Life Scale—old (WHOQL-OLD) according to Conrad et al. [26].

This tool is well validated for older adults from the age over 60 years and reflects the multidimensionality of the quality of life over different dimensions with so-called facets. The questionnaire consists of 24 multiple-choice questions, each with five response categories. The total quality of life score is formed from the individual facets. Six facets are distinguished in detail:

#### “Sensory abilities”

Central to our study, this facet generally captures patients’ assessment of their sensory functions (such as hearing, seeing, or tasting) [26].

#### “Autonomy”

The ability to live independently and self-determined is assessed by this facet [26].

#### “Past, present and future activities”

This facet captures achievements already made in life, current activities, and those planned for the future [26].

#### “Social participation”

Social participation in daily life as an essential factor for the quality of life is assessed here [26].

#### “Death and dying”

This facet includes attitudes and fears regarding one's death or the death of loved ones [26].

#### “Intimacy”

Interpersonal closeness and the importance of companionship are captured with this facet [26].

### Data analysis and statistical evaluation

Paper-based questionnaires were filled out by patients and subsequently organized using Microsoft Excel 2016 (Microsoft Corporation, Redmond, Washington). The statistic program GraphPad Prism Version 9 (GraphPad Software, Inc. San Diego) was used for statistical testing and generating graphic illustrations. Wilcoxon-matched-pairs test was used for comparison between two groups, and Kruskal–Wallis test was used to compare more than two groups. Boxplots were used for the graphical illustration. In addition to the median, the 25th and 75th percentiles are shown. All points outside the 5th and 95th percentile were presented as outliers as individual values. The significance level was assumed to be p ≤ 0.05.

## Results

The study included 31 patients (12 men and 19 women) aged between 71 and 92 years at the survey 6 years after the first CI treatment. The mean age was 79.1 ± 4.8 years. All participants had been treated with a CI unilaterally for the first time due to profound unilateral or bilateral hearing loss 6 years ago. Different implants of the manufacturer Cochlear™ (Cochlear: Cochlear Ltd., Macquarie, Australia) (10 × CI 512, 1 × CI 522, and 8 x CI 532) and MED-EL™ (MED-EL Elektromedizinische Geräte, Gesellschaft m.b.H., Innsbruck, Austria) (15 × Synchrony Mi1200) were used for the initial CI treatment. The average daily CI wearing time 6 years postoperatively was 10.5 ± 4.7 h.

Six patients were also implanted with a CI on the second side during the 6 years after the first CI treatment. In all bilaterally implanted patients, the second ear was implanted no more than 2.5 years after the first. For the second CI, different implants of the company Cochlear™ (2 × CI 512, 1 × CI 522, and 1 × CI 632) and MED-EL™ (2 × Synchrony Mi1200) were used.

### Freiburg monosyllabic speech test (FMS)

**Fig. 1:**
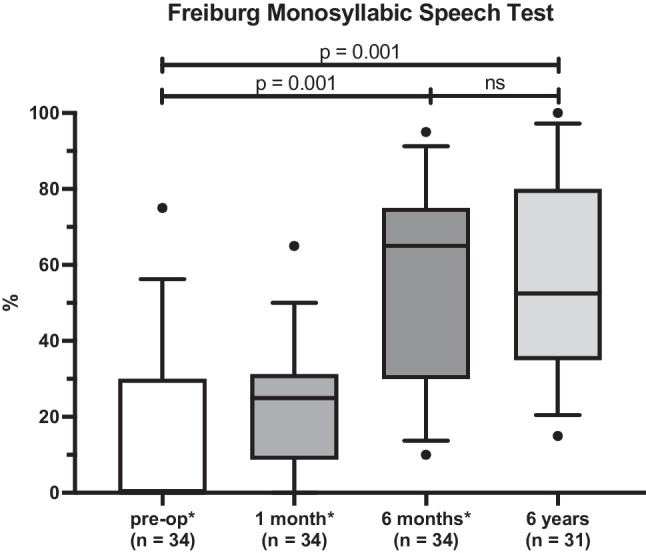
Freiburg Monosyllabic Speech Test (FMS). Results of FMS preoperatively, 1 month, 6 months of our previous study (*Issing et al. [19]) and six years postoperatively. Preoperative FMS was measured in the ear to be treated with a CI in best aided condition. After CI treatment the treated ear was assessed in CI-only condition

Six years postoperatively, patients’ monosyllable recognition score with the ear initially treated with a CI at 65 dB SPL averaged 59.5 ± 24.8%. There was no significant difference in FMS between the 6 month (56.5 ± 24.1%; [19]) and 6 year checkup (p = 0.341). From the preoperative measurement under best-aided conditions 14.7 ± 19.9% [19], the monosyllable recognition score increased significantly 6 years postoperatively (p = 0.001) (Fig. 1).

### World health organization quality-of-life scale—old (WHOQL-OLD)

#### WHOQL-OLD “Total score’’

**Fig. 2:**
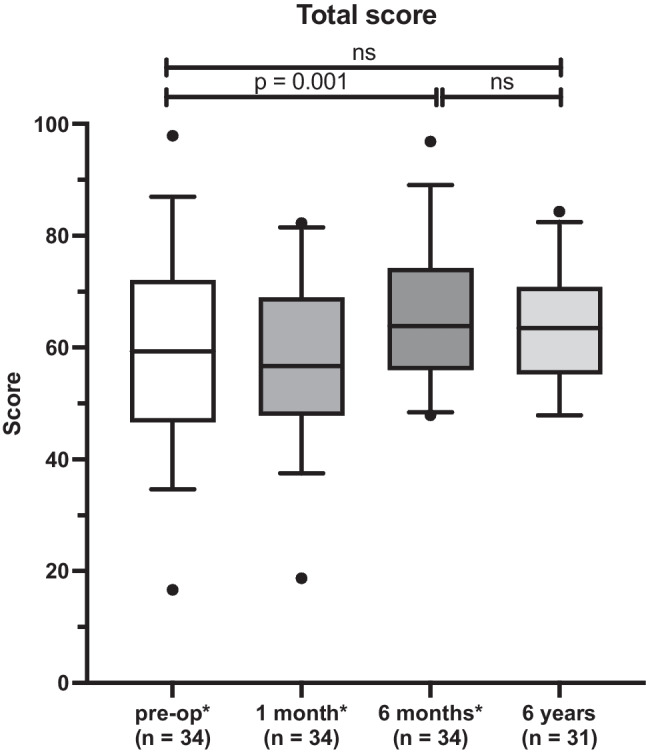
Total WHOQOL-OLD score. The total score is based on the six individual facets (see Fig. 3). In addition, the preoperative results as well as one and 6 months postoperatively of our previous study (*Issing et al. [19]) are shown

The "Ttotal score" after 6 years was 64.2 ± 10.6 points on average. No significant difference was found in the 6 month follow-up with 66.8 ± 2.2 points [19] (p = 0.12). There was no significant difference comparing the 6 year score with the preoperative score (60.0 ± 15.7 [19]) (p = 0.407) (Fig. 2; Table 1).

**Table 1:** Overview of total WHOQOL-OLD score and the individual facets

	Preoperative (n = 34) (Issing et al. [19])	1 month postoperative (n = 34) (Issing et al. [19])	6 months postoperative (n = 34) (Issing et al. [19])	6 years postoperative (n = 31)	Control Group ≥ 60 yrs (Conrad et al. [26])
Total score	60.0 ± 15.7	58.5 ± 13.6	66.8 ± 12.2	64.2 ± 10.6	68.0 ± 14.7
Sensory abilities	38.1 ± 22.6	29.2 ± 16.4	57.9 ± 12.6	56.8 ± 15.6	75.85 ± 21.1
Autonomy	63.2 ± 17.6	61.4 ± 17.0	65.3 ± 15.3	61.7 ± 12.7	68.9 ± 19.1
Past, present and future activities	66.2 ± 18.0	65.4 ± 15.4	68.4 ± 13.8	65.1 ± 13.0	65.34 ± 16.7
Social participation	61.04 ± 21.0	59.9 ± 18.0	70.6 ± 13.6	74.2 ± 14.4	69.0 ± 20.0
Death and dying	61.9 ± 30.0	65.4 ± 26.5	65.6 ± 25.1	57.5 ± 21.8	62.91 ± 24.3
Intimacy	69.3 ± 20.2	69.9 ± 21.0	73.0 ± 16.3	69.8 ± 15.5	65.81 ± 20.9

Additional are the data preoperatively, one and 6 months postoperatively of our previous study (Issing et al. [19]). Values of an age-matched control group according to Conrad et al. [26] are displayed

#### WHOQL-OLD “Sensory abilities’’

**Fig. 3:**
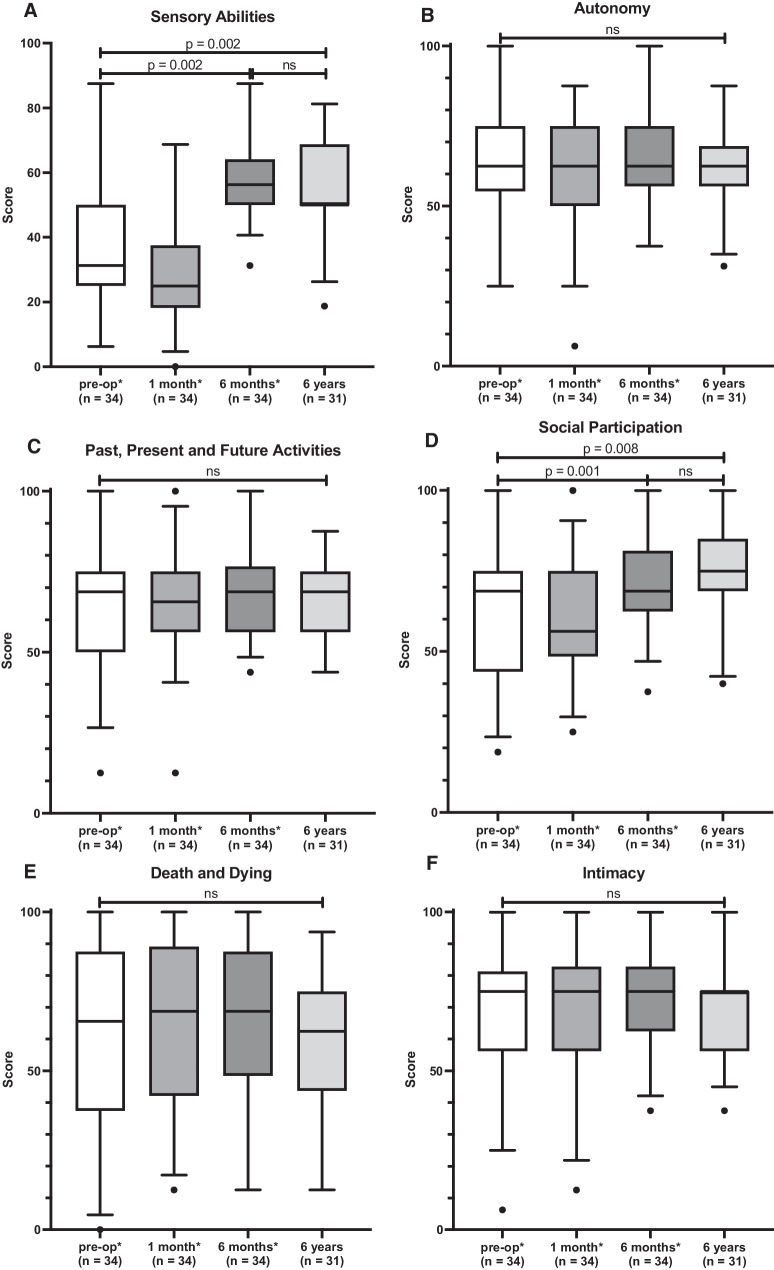
Facets of WHOQOL-OLD. In (**A**–**F**), the individual facets of WHOQOL-OLD are shown. In addition, the preoperative results as well as one and 6 months postoperatively of our previous study (*Issing et al. [19]) are presented. **A** “Sensory abilities”; **B** “Autonomy”; **C** “Past, present and future Activities”; **D** “Social participation”; **E** “Death and dying”; **F** “Intimacy”. There were no significant differences in any facet between the follow-up 6 months and 6 years postoperatively

For the facet “Sensory Abilities”, the mean score 6 years postoperatively was 56.8 ± 15.6. There was no significant difference compared to the control 6 months postoperatively (57.9 ± 12.6 [19]) (p = 0.816). From the preoperative assessment (38.1 ± 22.6 points [19]) to the 6 year control, there was a significant increase (p = 0.002) (Fig. 3; Table 1).

#### WHOQL-OLD “Autonomy’’

The average score for “Autonomy” 6 years postoperatively was 61.7 ± 12.7 points. A non-significant decrease was observed compared to the 6 month control (65.3 ± 15.3 [19]) (p = 0.074). Overall, there was no significant change throughout the study period (p = 0.866) (Fig. 3; Table 1).

#### WHOQL-OLD “Past, present, and future activities”

Six years postoperatively, the mean score was 65.1 ± 13.0 points. 6 months postoperatively, the score was 68.4 ± 13.8 [19]. The difference was not significant (p = 0.162). Overall, there was no significant change during the entire study period (p = 0.731) (Fig. 3; Table 1).

#### WHOQL-OLD ‘‘Social participation’’

For “Social participation”, the score at 6 years postoperatively was 74.2 ± 14.4 points. There was no significant difference to the 6 month control 70.6 ± 13.6 [19] (p = 0.224), but there was a significant difference to the preoperative measurement (61.4 ± 21.0 points [19]) (p = 0.008) (Fig. 3; Table 1).

#### WHOQL-OLD “Death and dying”

Six years after the initial unilateral CI treatment, the mean score was 57.5 ± 21.8 points. When compared to the 6 month control (65.6 ± 25.1 points [19]), there was no significant decrease (p = 0.282). There were no significant changes over the entire study period (p = 0.36) (Fig. 3; Table 1).

#### WHOQL-OLD ‘‘Intimacy’’

For the facet “Intimacy”, the average score after 6 years was 69.8 ± 15.5 points. In the 6 months postoperative control (73.0 ± 16.3 points [19]), there was no significant decrease (p = 0.382). Over the entire 6 year period, there was no significant change (p = 0.881) (Fig. 3; Table 1).

## Discussion

Over the last decades, the treatment of deaf and severely hearing-impaired patients of all age groups with a CI has become a routine procedure with excellent results in speech understanding for suitable candidates [8, 10, 13, 14, 22, 27, 28]. In recent years, the proportion of CI candidates over the age of 65 has been steadily increasing, with comparable results in speech understanding to younger patients [7, 8]. In recent years, the evaluation of the quality of life has become an integral part of the assessment of treatment success in Germany and is recommended in the German Cochlear Implant Guideline and the German Whitebook on Cochlear Implant Care. These recommendations also form the basis for the new German Cochlear Implant Registry and are a prerequisite for certification as a Cochlear Implant Center in Germany [15–18].

In our previous study, we also demonstrated a rapid and significant improvement in monosyllabic discrimination within 6 months postoperatively (p = 0.001). The monosyllabic discrimination was stable from the control 6 months postoperatively to the 6 year control (p = 0.341). Our data demonstrate not only a very rapid but also stable improvement in monosyllabic discrimination over the years in elderly patients.

Hearing impairment is a particular risk factor for elderly patients due to the specific psychosocial situation with often increasing physical limitations and resulting in a reduction of social participation [4, 6]. The return to “acoustic social life” due to hearing rehabilitation with CI treatment is, therefore, the basis of improving quality of life and should thus have a central focus in the clinical outcome assessment. Besides the health-related quality of life, such as the widely used Nijmegen Cochlear Implant Questionnaire, general quality-of-life questionnaires are available. As the aim of rehabilitation is, whenever possible, to reach the outcomes of a comparable healthy population, we used the World Health Organization Quality-of-Life Scale -old (WHOQL-OLD) questionnaire to allow the comparison with a healthy age-matched general population [14, 20, 21, 24, 26]. In addition, this questionnaire has been validated specifically for patients over the age of 60. For the total score, as well as for all six facets, there were no significant changes from the control 6 months postoperatively to 6 years postoperatively. However, when comparing the total score from the preoperative measurement to the control 6 years postoperatively, there is no significant difference (p = 0.407) either. To explain these results, the individual facets have to be examined in more detail: The facets “Autonomy” (p = 0.074) and “Past, present and future activities” (p = 0.162) showed a non-significant decrease but clear negative trend in the score from the survey 6 months postoperatively to 6 years postoperatively. In particular, the overperformers decreased. Since the facets “Sensory abilities” (p = 0.816), “Social pParticipation” (p = 0.224), as well as the monosyllabic discrimination (p = 0.342) did not change significantly during this period, other age-related factors not associated with hearing can be assumed as cause.

In monosyllabic discrimination, there was even a slight non-significant increase from the 6 month to the 6 year control. This not only confirms the suspected other factors for the decrease of the total score compared to the 6 year follow-up but also gives evidence for an continuous improvement of the monosyllabic discrimination even after more than 6 months postoperatively in elderly patients. Thus, not only do the poor performers but also the top performers improve further.

To evaluate the results of our study 6 years after hearing rehabilitation with CI, we compared the results to a healthy elderly population, according to Conrad et al. [26] (Table 1). However, the results must be considered in the context of the Conrad et al. cohort being slightly younger, with a mean of 71.3 ± 8.3 years compared to our cohort with 79.1 ± 4.8 years. Regarding the total score, our cohort with a score of 64.2 ± 10.6 does not reach the values of the healthy general population 68.0 ± 14.7 (p = 0.057) even 6 years postoperatively. However, this can also be partially explained by the older age of our cohort and presumably more age-related other infirmities. A closer look at the individual facets reveals significant differences compared to the healthy general population, particularly in the “Sensory abilities” (p = 0.001). The same is true for “Autonomy” (p = 0.001). Thus, although hearing rehabilitation with CI leads to a significant improvement in monosyllabic discrimination, the affected patients assess the performance of their sensory functions significantly worse than the general population. This may be due to a poorer speech comprehension compared to healthy persons despite the CI, but also to persisting everyday problems, especially with hearing in noise. This clearly demonstrates that there is a relevant difference from CI users to the normal hearing. This illustrates the need for further improvement of the CI technology. Possibly also new therapeutic strategies such as optical cochlear implants may help to “close the gap” in the future [29].

Social participation ultimately represents the success of CI treatment that can be directly experienced by each patient. Through hearing rehabilitation with CI, the social deprivation that most elderly hearing impaired patients suffer can usually be successfully reversed [30]. Our data not only indicate stability of “social participation” after hearing rehabilitation for many years, but as the only facet “Social participation” increases—however not significantly—from the control 6 months postoperatively with 70.6 ± 13.6 points to 6 years postoperatively with 74.2 ± 14.4 points (p = 0.1). Interestingly, our study cohort performed better than the general population both at 6 months and 6 years postoperatively (69.0 ± 20.0; p = 0.1).

A critical view on our study reveals potential limitations. Although this is a prospective longitudinal study with a small loss of follow-up of only three patients (8.8%), it is not a randomized controlled study. For ethical reasons, a randomized controlled trial design was not possible. Instead of a control group, a healthy German elderly population, according to Conrad et al. [26], was used. Although this control group is suitable in principle, the data must be considered in the context that the cohort of Conrad et al. [26] is on average almost 8 years younger. As a result, comparisons with the general population according to Conrad et al. are slightly too stringent, as usual age-related changes tending to decrease the quality of life of an older cohort are not considered. Consequently, in addition to the cohort treated with a CI, an age-matched healthy control group would be preferable, that would have to be examined at the same time points. Due to the lack of a sufficient number of healthy age-matched patients, this approach was not feasible.

Ultimately, it is not only the audiological measurable success but rather the return to social life and thus the improvement in quality of life that defines the CI-treatment success experienced by the patient. In summary, our study data indicate a significant increase in “Social participation” in addition to a rapid and stable improvement in monosyllabic discrimination over 6 years. As the only facet, the “Social participation” score continues to increase slightly on average over the years, even better than the average population. As a consequence, in particular elderly CI candidates, should not only be counseled for improvement in speech understanding, but also on the multidimensional positive impact especially on social participation. In addition, a continuous monitoring of the quality of life should be performed routinely during the CI follow-up in order to be able to assess this dimension of the CI-treatment success.

## Conclusion

Our study data demonstrate a rapid and stable improvement in quality of life over the 6 years in elderly patients over the age of 65. Moreover, the importance of the systematic assessment of the quality of life, as included in the German Cochlear Implant Guideline and the German Whitebook on Cochlear Implant Care as a central treatment success parameter, is underlined [15–18]. In particular, “Social participation” as a treatment success directly experienced by the patient reaches the level of the general population within only 6 months postoperatively and even exceeds this level 6 years after CI treatment. Hearing rehabilitation with a CI can be explicitly recommended in case of audiological indication without an age limit. The multidimensional positive effects of CI treatment in elderly patients should also be discussed in detail during preoperative consultation.
